# Younger age doubles medium‐term revision risk after total knee arthroplasty: A nationwide multicenter cohort of 5980 knees from the French SCORE I registry

**DOI:** 10.1002/ksa.70165

**Published:** 2025-11-04

**Authors:** Alessandro Carrozzo, Émilie Bérard, Ophélie Manchec, Stefano Jacotti, Régis Pailhe, Etienne Cavaignac

**Affiliations:** ^1^ Dipartimento di Scienze della Vita della Salute e delle Professioni Sanitarie, Università degli Studi ‘Link Campus University’ Rome Italy; ^2^ Department of Clinical Epidemiology and Public Health, CERPOP INSERM‐University of Toulouse III, Toulouse University Hospital (CHU) Toulouse France; ^3^ Department of Orthopaedic Surgery Hôpital Pierre Paul Riquet, CHU de Toulouse Toulouse France; ^4^ Clinique Aguilera Ramsay Santé Biarritz Biarritz France

**Keywords:** age factors, octogenarian, registries, revision arthroplasty, total knee arthroplasty, treatment outcome

## Abstract

**Purpose:**

Although age is a known predictor of outcomes following total knee arthroplasty (TKA), most large‐scale studies rely on registry data that lack clinical detail and combines multiple implant designs. This study aimed to determine whether age has an independent effect on implant survivorship and clinical outcomes when a single TKA design is used in a national cohort.

**Methods:**

A multicenter retrospective cohort analysis was performed using the Amplitude® registry, which prospectively recorded every primary TKA done with the same implant across 15 healthcare facilities between 2002 and 2022. Patients were grouped a priori into three age brackets: <65 years, 65–79 years and >79 years. The primary endpoint was revision‐free survival; the secondary endpoints were surgery‐free survival, the International Knee Society Score score and knee flexion at one and five years postoperative. Survival analyses were adjusted for sex, body mass index (BMI), American Society of Anesthesiologists (ASA) grade, fixation type and navigation use.

**Results:**

Of the 5980 TKAs included, 1276 were in patients <65 years, 3589 in those 65–79 and 1115 in patients >79. The 5‐year revision‐free survival was 97.5% (<65 years), 98.9% (65–79 years) and 98.7% (>79 years). Compared to the 65–79 year‐old group, patients <65 had twice the risk of revision (adjusted hazard ratio [HR] 2.03; 95% confidence interval [CI] 1.20–3.43; *p* = 0.008), while no difference was found for those >79 (HR 1.14; *p* = 0.728). Surgery‐free survival and complications followed similar trends. No interaction between age and sex, BMI, ASA, fixation or navigation was observed. Functional outcomes improved across all ages; younger patients had higher KSS, but age had no effect on postoperative flexion.

**Conclusions:**

Age is an independent predictor of implant survivorship following TKA. While younger patients have good functional outcomes, they remain the Achilles heel of TKA due to their higher risk of revision, whereas patients >79 years of age have comparable implant longevity to 65–79‐year‐old patients.

**Level of Evidence:**

Level III retrospective comparative study.

**Abbreviations**
ASA: American Society of Anesthesiologists (physical status classification)
BMI: body mass index
CI: confidence interval
HR: hazard ratio
KSS: International Knee Society Score
TKA: total knee arthroplasty

## INTRODUCTION

Total knee arthroplasty (TKA) has become the gold standard solution for end‐stage knee osteoarthritis, with more than 110,000 primary procedures performed annually in France and numbers projected to rise steadily over the next two decades [3, 10]. This is in part due to an overall expansion of indications to 'non‐traditional' age groups at both ends of the spectrum [28, 29].

Patients younger than 65 years and those over 80 years already represent roughly one‐fifth of all TKAs performed each year [9, 15]. However, different studies and registry data consistently show that younger patients have a two‐ and five‐fold higher risk of revision than patients in their 60s and 70s, largely because of activity‐related aseptic loosening, instability and early infection [6, 12, 22]. Conversely, patients over 80 have more medical and surgical complications, with worse clinical outcomes than younger patients, but a comparable or even higher TKA survival rate when they live beyond the first postoperative year [9, 16, 30]. This study was done to address limitations in the current age‐related TKA evidence, which is largely derived from registries that mix implant designs and lack clinical detail, or from single‐centre cohorts that are underpowered for interaction testing. Consequently, the independent influence of age on implant longevity is still unclear. Therefore, this study was designed to combine the scope of a registry with the detail level of a cohort by analysing a large national registry featuring a single implant system that collected comprehensive clinical data for each patient. The primary aim was to compare revision‐free survivorship across three predefined age brackets, while adjusting for potential confounders. The secondary aims were to compare all‐cause revision rates and functional outcomes. It was hypothesised that

1. Patients <65 years have higher risks of revision and re‐operation than those 65–79 years, independent of sex, body mass index (BMI), American Society of Anesthesiologists (ASA), fixation type and navigation use;
2. Patients >79 years have implant survivorship comparable to those 65–79 years but with slightly lower knee‐specific outcome scores;
3. No clinically relevant interactions exist between age and sex, BMI, ASA, fixation or navigation.

## METHODS

A retrospective study was carried out on a national, prospectively maintained registry managed by Amplitude® that has recorded every primary TKA done with the same ultra‐congruent, posterior cruciate‐sacrificing, rotating‐platform deep‐dish mobile bearing design in 15 high‐volume orthopaedic departments since March 2002.

### Participants

Patients were eligible when the indication was primary or secondary osteoarthritis. Patients were excluded if the file mentioned inflammatory arthritis (*n* = 114), tumour reconstruction (*n* = 2) or did not have age listed (*n* = 120), for a total of 236 exclusions.

Patients were stratified a priori by chronological age into three groups: <65, 65–79 and >79 years, to contrast ‘young working‐age’, ‘standard’ and ‘octogenarian’ recipients. These cut‐off points reflect evidence that patients <65 have a higher revision risk, whereas octogenarians achieve survivorship comparable to (or exceeding) younger cohorts but with lower functional outcomes [8, 9, 12, 17, 22, 26, 30].

### Variables and outcome measures

The registry captured baseline (sex, BMI, ASA, International Knee Society Score [KSS] and knee flexion), intraoperative (surgical approach, fixation/cementation strategy, computer navigation use, patellar management, tourniquet use), and follow‐up data (complications, revisions/reoperations, goniometer‐measured active flexion, KSS) [11].

The primary endpoint was revision‐free survival, defined as the time elapsed from the index TKA to first component exchange or removal; the reason for revision was recorded. Secondary endpoints were surgery‐free survival (any reoperation), KSS and knee flexion at 1 and 5 years. For time‐to‐event analyses, patients contributed person‐time from the index procedure to the earliest of revision/reoperation, death, or last contact. Patients with only one postoperative visit were censored at that visit if event‐free.

### Surgical procedure

All TKAs were implanted through a medial parapatellar arthrotomy or a lateral parapatellar approach (usually for valgus deformities). Fixation methods were left up to the surgeon and included cementless implants or fully and hybrid cementation; a press‐fit tibial stem was frequently added in obese patients or those with severe deformity. Computer navigation use and patellar resurfacing was left to the discretion of the surgeon.

The registry and this analysis complied with the French data‐protection authority (Commission Nationale de l'Informatique et des Libertés [CNIL]) MR‐004 standard. Authorisation was granted under CliniRecord No. 1355265, and data storage and processing was registered in the national Health Data Hub (No. F20210913151920) by Amplitude®. Because this was a registry‐based cohort, no sample size calculation was performed.

### Statistical analysis

Before analyses, verification of missing or aberrant or inconsistent data was conducted. After corrections, the database was locked. The analysis was performed on the locked database. Patient characteristics were summarised using the appropriate descriptive statistics depending on the type of variables. Descriptive statistics included mean with SD for continuous variables, and number of non‐missing observations with frequency (%) for categorical variables. Categorical variables were compared between the three groups using the *χ* ^2^ test (or Fisher's exact test when necessary). The analysis of variance (ANOVA) (or Kruskal‐Wallis test if necessary) was used to compare the distribution of continuous variables.

To analyse survivorship, Kaplan–Meier survival curves were calculated together with 95% confidence intervals (CI) and compared using the log‐rank test together with a non‐adjusted Cox model first, and then using a Cox model adjusted for sex, BMI, ASA, navigation use and cementation. All interactions between age and sex, BMI, ASA, navigation or cementation were tested in the models.

All reported *p*‐values were two‐sided, and the significance threshold was set at < 0.05. Statistical analyses were performed using STATA software 18.0 (STATA Corp., College Station, TX, USA).

## RESULTS

### Patient characteristics

**Figure 1:**
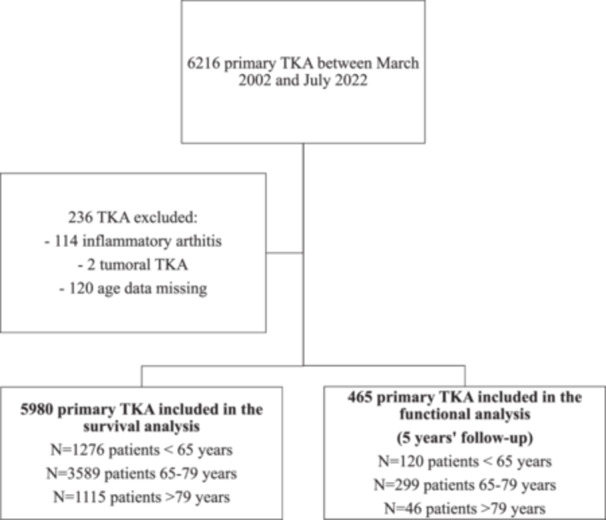
Flowchart of the included patients. TKA, total knee arthroplasty.

Between March 2002 and July 2022, 5980 primary TKA procedures met the eligibility criteria. Patients were stratified into three age brackets: <65 years (*n* = 1276, 21%), 65–79 years (*n* = 3589, 60%) and >79 years (*n* = 1115, 19%; Figure 1).

Younger patients were more frequently male, heavier (mean BMI 31.7 kg/m²) and healthier (78% ASA I and II), whereas octogenarians were predominantly female, lighter (mean BMI 28.2 kg/m²) and had a higher comorbidity burden (31% ASA III–V; Table 1). The rate of navigation use, around 60%, was similar no matter the patient age, while the proportion of fully cemented or hybrid fixation increased with age and preoperative KSS values were lower with increasing age (Table 1).

**Table 1:** Baseline demographics and surgical characteristics by age bracket.

	<65 years (*n* = 1276)	65–79 years (*n* = 3589)	>79 years (*n* = 1115)	*p* value
Female sex, *n* (%)	692 (55.6)	2196 (62.6)	741 (68.2)	<0.0001
BMI, mean ± SD (kg/m²)	31.7 ± 6.2	30.1 ± 5.1	28.2 ± 4.4	<0.0001
ASA III–V, n (%)	244 (22.1)	838 (26.4)	314 (31.2)	<0.0001
Cemented/hybrid fixation, *n* (%)	256 (20.1)	898 (25.0)	352 (31.6)	<0.0001
Navigation‐assisted TKA, *n* (%)	766 (60.0)	2 135 (59.5)	660 (59.2)	0.910
Preop KSS, mean ± SD	100.3 ± 21.2	97.3 ± 21.2	89.3 ± 20.8	<0.0001

Abbreviations: ASA, American Society of Anesthesiologists; BMI, body mass index; KSS, International Knee Society Score; TKA, total knee arthroplasty.

### Primary endpoint—Implant survivorship

**Figure 2:**
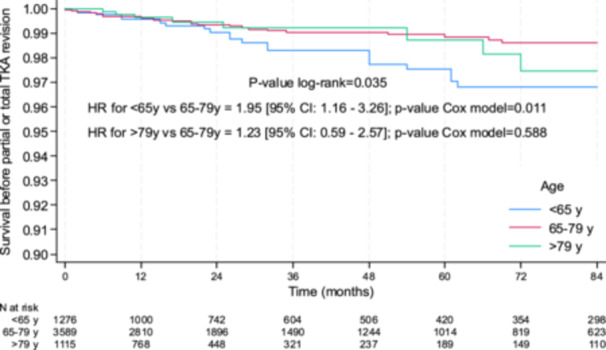
Kaplan–Meier plot of TKA survivorship free from any component revision. CI, confidence interval; HR, hazard ratio; TKA, total knee arthroplasty.

With a median follow‐up of 24 months, 68 revisions were recorded (1.1%). The 5‐year Kaplan–Meier survivorship free from any component revision was 97.5% ( <65 years), 98.9% (65–79 years) and 98.7% (>79 years; Table 2 and Figure 2).

**Table 2:** Survivorship free from revision and Cox hazard ratios (HR), with and without adjustment for sex, BMI, ASA class, navigation and fixation method.

Age group	5‐Year survival (95% CI)	*p* value (log rank)	HR vs. 65–79 years (95% CI)	*p* value (Cox model)	Adjusted HR vs. 65–79 years (95% CI)	*p* value (Cox model)
<65 years	97.5% (96.0–98.5)	0.035	1.95 (1.16–3.26)	0.011	2.03 (1.20–3.43)	0.008
65–79 years	98.9% (98.3–99.3)		Reference	–	Reference	–
>79 years	98.7% (96.8–99.5)		1.23 (0.59–2.57)	0.588	1.14 (0.54–2.41)	0.728

Abbreviations: ASA, American Society of Anesthesiologists; BMI, body mass index; CI, confidence interval.

Compared with the 65–79 reference group, the <65 group had almost twice the risk of revision (*p* = 0.011), while the risk was not different for the >79 group (*p* = 0.588). Adjusting for sex, BMI, ASA class, navigation and fixation method did not change the estimates and no interaction between age and these covariates were statistically significant. The reasons for revision differed between age groups (*p* = 0.016; Table 3).

**Table 3:** Reasons for total knee arthroplasty revision by age bracket.

Reason for revision	<65 years (*n* = 1276)	65–79 years (*n* = 3589)	>79 years (*n* = 1115)	Total (*n* = 5980)	*p* value
Aseptic loosening, *n* (%)	6 (0.47%)	7 (0.20%)	2 (0.18%)	15 (0.25%)	0.016
Deep infection, *n* (%)	9 (0.71%)	18 (0.50%)	3 (0.27%)	30 (0.50%)	
Periprosthetic fracture, *n* (%)	1 (0.08%)	2 (0.06%)	2 (0.18%)	5 (0.08%)	
Arthrofibrosis/stiffness, *n* (%)	5 (0.39%)	2 (0.06%)	0 (0.00%)	7 (0.12%)	
Unexplained pain, *n* (%)	5 (0.39%)	3 (0.08%)	1 (0.09%)	9 (0.15%)	
Dislocation/instability, *n* (%)	0 (0.00%)	1 (0.03%)	1 (0.09%)	2 (0.03%)	
Total revisions, *n* (%)	26 (2.04%)	33 (0.92%)	9 (0.81%)	68 (1.14%)	

### Secondary endpoints

#### Overall surgery‐free survival

**Figure 3:**
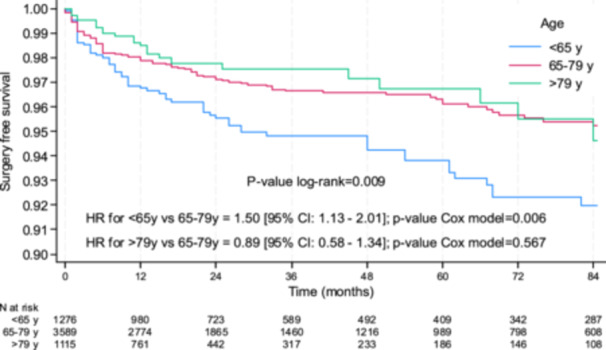
Kaplan–Meier plot for overall surgery‐free survival after total knee arthroplasty. CI, confidence interval; HR, hazard ratio.

Including all re‐operations (debridement for infection, fracture fixation, mobilisation under anaesthesia and patellar procedures), there were 225 events (3.8%). The 5‐year surgery‐free survival was 93.8%, 96.1% and 96.7% in the <65, 65–79 and >79 groups, respectively (Table 4 and Figure 3).

**Table 4:** Surgery‐free survival (all causes) and Cox hazard ratios (HR), with and without adjustment for sex, BMI, ASA class, navigation use and fixation method.

Age group	5‐year survival (95% CI)	*p* value (log rank)	HR vs. 65–79 years (95% CI)	*p* value (Cox model)	Adjusted* HR vs. 65–79 years (95% CI)	*p* value (Cox model)
<65 years	93.8% (91.9–95.3)	0.009A	1.50 (1.13–2.01)	0.006	1.51 (1.13–2.03)	0.006
65–79 years	96.1% (95.2–96.8)		Reference	–	Reference	–
>79 years	96.7% (94.7–98.0)		0.89 (0.58–1.34)	0.567	0.84 (0.55–1.29)	0.432

Abbreviations: ASA, American Society of Anesthesiologists; BMI, body mass index; CI, confidence interval.

Compared with the 65–79 reference group, the <65 group had almost twice the risk of surgery (*p* = 0.006), while the risk was not different for > 79 group (*p* = 0.567). Adjustment for sex, BMI, ASA class, navigation use, and fixation method did not change the estimates and no interaction between age and these covariates were statistically significant.

#### Functional outcomes and range of motion

At 1 year postoperative, the mean KSS exceeded 180 points in the two younger cohorts and was 176 points in those >79 years (*p* < 0.0001; Table 5). At 5 years postoperative, the KSS still differed similarly by age (*p* = 0.028). This clinically relevant difference in the >79 group was due to KSS‐knee score; the KSS‐function score was not significantly different between age groups. Postoperative knee flexion averaged 115°–120° at 1 and 5 years, with no significant age effect.

**Table 5:** Functional outcomes by age group.

	Time	<65 years	65–79 years	>79 years	*p* value
Flexion (°), mean ± SD	1 year	115.0 ± 12.6	116.3 ± 11.0	116.3 ± 10.0	0.086
Flexion (°), mean ± SD	5 years	115.8 ± 13.0	118.3 ± 11.5	120.2 ± 10.5	0.248
KSS Total, mean ± SD	1 year	182.7 ± 19.5	182.4 ± 19.2	176.2 ± 20.1	**<0.0001**
KSS Total, mean ± SD	5 years	183.0 ± 23.9	181.4 ± 20.0	175.0 ± 20.1	**0.028**
KSS Function, mean ± SD	1 year	91.4 ± 10.9	92.6 ± 9.3	92.9 ± 9.0	0.1069
KSS Function, mean ± SD	5 years	91.8 ± 13.7	93.3 ± 8.9	94.4 ± 6.3	0.9422
KSS Clinical, mean ± SD	1 year	91.3 ± 12.0	90.0 ± 13.3	83.2 ± 15.2 ±	**<0.0001**
KSS Clinical, mean ± SD	5 years	91.3 ± 13.9	88.1 (14.4)	80.6 ± 17.0	**0.0016**

*Note*: Knee flexion (degrees) and Knee Society Score (KSS) Total, Clinical, and Function scores are reported as mean ± SD. *p* Values are for between‐group comparisons across age brackets (<65, 65–79, >79). The MCID is 5 points for the KSS Total and 6 and 7 for the Function and Clinical subscales, respectively [19, 23]. Boldface indicates significant difference.

Abbreviation: MCID, minimal clinically important difference.

## DISCUSSION

The most important finding of this study was that chronological age affected clinical outcomes after primary TKA: patients <65 years had a two‐fold higher risk of revision and re‐operation, whereas the survivorship in octogenarians (>79 years) was equivalent to the standard age group (65–79 years). However the complication types were different and the functional scores lower.

The revision‐free survival for the <65 cohort (97.5% at 5 years) is in line with registry and single‐centre study that consistently show inferior implant longevity in younger recipients. In 6275 consecutive TKAs, McCalden et al. found that younger patients had slightly better WOMAC and KSS gains but had significantly lower implant survivorship (5‐/10‐year: 95.5%/92.2% vs. 98.1%/97.6% in patients >70 years). Infection was the leading cause of failure across all ages, with aseptic loosening and instability happening more frequently in younger patients [21]. Furthermore, a systematic review of 1283 TKAs in patients <55 years reported substantial functional improvement but a 5.4% cumulative all‐cause revision rate at a mean 10.8‐year follow‐up [5].

Recent Italian and German registry analyses confirm that expanding TKA indications to patients less than 65 years is associated with higher medium‐ and long‐term failure risk. In 45,488 TKAs, Perdisa et al. showed age‐graded declines in 15‐year survivorship, 78.7% (<50), 89.4% (50–65), 94.8% (66–79), with the failure risk being 3.1× (<50) and 1.8× (50–65) relative to the 66–79 bracket [26]. In nearly 301,000 arthroplasties, Straub et al. found a significantly higher aseptic revision rate in patients <65 (*p* < 0.0001); the 7‐year cumulative rate was 5.0% (<65) vs. 2.9% (65–74) and 2.4% (>74). In unconstrained TKA, higher Elixhauser scores increased risk across ages [30].

In the current study, the adjusted hazard ratio of 2.03 for revision (in <65 vs. 65–79) is in line with those observations and provides further evidence that chronological age remains an independent predictor even after controlling for BMI, ASA class, fixation technique and navigation use.

Several mechanisms may contribute to the more frequent failures in younger patients: higher activity level and mechanical demands (often with higher BMI), increased aseptic loosening, instability, and polyethylene wear [2, 7, 13]. Also, the greatest absolute number of septic revisions was observed in the high‐volume 65–79 cohort, but the proportional contribution of infection remained dominant across ages, as reported earlier [21]. Younger patients may be more demanding and consequently less tolerant of residual pain or stiffness, accelerating the decision to revise, an effect highlighted by patient‐reported dissatisfaction studies [1, 27].

In octogenarians, lower functional demands likely reduce polyethylene wear and soft‐tissue stress. Patients >79 years had a 5‐year survivorship of 98.7% with no excess revision hazard versus those 65–79 years, consistent with contemporary studies. Deroche et al. similarly found no survivorship difference at ≥5 years with a modern cemented posterior‐stabilising design, despite slightly lower KSS function in older adults; overall knee scores and range of motion were comparable [9]. In a matched retrospective study of 206 TKAs, Andreozzi et al. reported significant functional gains in both octogenarians and younger patients, with comorbidities, not age, leading to postoperative complications. Octogenarians had higher transfusion risk (OR 3.4) and longer hospital stay ( + 1.7 days), but age itself was not an independent predictor for most complications [4]. A systematic review of 13 comparative studies by Courage et al. confirmed higher complication rates in patients >79 years (surgical up to 21%, medical up to 17%), and higher 90‐day mortality (0%–2%) versus younger cohorts, while wound complications and PROMs showed wide variability and inconsistent age‐related trends [8]. Klasan et al. reported comparable implant survivorship to ‘average‐age’ patients despite higher rates of medical complications and mortality in octogenarians, with slightly but significantly lower PROMs [14]. Consistent with these published study, the current study found better PROMs in younger patients. Moreover, a systematic review indicates PROMs are generally highest when TKA is performed in the seventh decade, with greater revision risk in younger patients and increasing mortality in older patients [16].

In the current study, infection was the leading cause of revision across all age groups, consistent with national registry patterns from Sweden and Australia [17, 31]. Aseptic loosening was more frequent in younger patients (0.47% vs. 0.20% and 0.18% in older groups), consistent with McCalden's findings of higher loosening rates in younger patients [21]. A systematic review of patients ≤55 years reported 5–10 year survivorship ≥90%, with wear/loosening and infection as the most common reasons for revision [25]. In the current study, no clinically relevant interaction was detected between age and sex, BMI, ASA grade, navigation or fixation method on the survival endpoints.

Cemented components are usually favoured in older adults and are sometimes seen as protective, while the best fixation method in younger patients still being debated. In a multicenter cohort, Manchec et al. found equivalent 5‐year survivorship between cemented and cementless TKA, with similar aseptic reoperation rates and no medium‐term differences in functional outcomes [20]. A large registry analysis of 115,177 TKAs in patients <65 years reported a 10‐year survivorship >92% for cemented and hybrid fixation, while uncemented TKAs had a higher revision risk early and late, supporting cemented fixation as the current standard in younger patients [24]. In a multi‐registry meta‐analysis (>1 million TKAs), Lewis et al. found better survivorship in patients ≥65 years, but the association with fixation type was inconsistent across data sets [18].

### Strengths and limitations

The study's strengths are that it analysed a large multicenter cohort of patients who had all undergone TKA with a single implant design, the database had collected clinical covariates prospectively, time‐to‐event analyses were done and a large share of octogenarians were included. Its limitations include the retrospective design, limited generalisability because only one TKA implant was studied, a median follow‐up of 24 months (although time‐to‐event analysis enabled 5‐year estimates, and 25% of patients had ≥63 months of follow‐up), incomplete 5‐year PROMs, and changing perioperative practices over the inclusion period. These findings best reflect early to medium‐term hazards; later aseptic failures will require longer follow‐up.

## CONCLUSIONS

Age acts as an independent, non‐modifiable, prognostic factor. Young working‐age patients remain the Achilles heel of TKA, with a revision risk that is almost double that of older patients, despite better functional outcomes. Conversely, octogenarians had implant longevity comparable to ‘standard‐age’ TKA recipients. TKA survivorship is stable across sex, BMI, ASA status, navigation use and fixation method. In practice, these data support close early surveillance and tailored counselling for younger TKA recipients and suggest that advanced age alone should not preclude TKA when a patient's comorbidities can be optimised.

## AUTHOR CONTRIBUTIONS

All authors contributed to the study. Émilie Bérard and Régis Pailhe have ideated the study and established the study design. Material preparation, data collection and analysis were performed by Alessandro Carrozzo, Émilie Bérard, Ophélie Manchec and Stefano Jacotti. The first draft of the manuscript was written by Alessandro Carrozzo, and Émilie Bérard, Émilie Bérard, Ophélie Manchec, Stefano Jacotti and Régis Pailhe had substantially edited the draft. All authors read and approved the final manuscript.

## CONFLICT OF INTEREST STATEMENT

Etienne Cavaignac: Consultant for Arthrex, Amplitude and Biobank. The remaining authors declare no conflicts of interest.

## ETHICS STATEMENT

The use of the database was conducted under the authorisation of the CNIL, registered in CliniRecord under the No. 1355265. Amplitude® has registered the data for the long‐term evaluation of the SCORE prosthesis on the public platform ‘Health Data Hub’ under number No. F20210913151920. All data used in this study are sourced from this registry, which we managed according to the CNIL standard methodology MR‐004.

## Data Availability

The data sets generated and analysed during the current study are not publicly available but are available from the corresponding author on reasonable request.
